# Sustainability in swine production

**DOI:** 10.1093/af/vfac081

**Published:** 2022-12-14

**Authors:** John Scott Radcliffe, James L Sartin

**Affiliations:** Department of Animal Sciences, Purdue University, West Lafayette, IN 47907, USA; American Society of Animal Science, Champaign, IL 61822, USA

**Keywords:** environment, health, society, sustainability, swine

Sustainability is an often-hailed concept in animal agriculture and indeed all areas of human endeavor. Unfortunately, we often miss the mark in discussing sustainability by seemingly looking for a single solution to a large and varied problem. To solve issues of sustainability in swine production, animal agriculture and across all human endeavors will take more than the single superhero: Sustainability Swine, depicted on the front cover of this issue of *Animal Frontiers*. In this issue of *Animal Frontiers*, we endeavored to present the myriad of opportunities and challenges associated with obtaining sustainability in swine production with particular emphasis on where swine production has made strides enhancing sustainability and what must be addressed in the near future.

This issue concentrates on sustainability in swine production, but the importance of this topic to the Animal Sciences can be seen through the previous coverage provided by this journal. The most recent issue dedicated to sustainability was compiled by Dr. Kristen Johnson who served as a guest editor in 2021 on an issue entitled “Charting a course for sustainability” (Johnson, 2021). This issue examined the course of sustainability in animal agriculture throughout the world. This included sustainability in animal science in South Africa and greenhouse gas emissions (Casey, 2021), the development of market based tools for accelerating cattle sustainability in Canada (Hougen-Kozyra, 2021), changes in New Zealand red meat production (Moot and Davison, 2021), sustainability of ruminant livestock production in Ireland (O’Mara et al., 2021), low carbon and environmentally friendly livestock: The Costa Rican approach (Montenegro and Abarca, 2021), livestock sustainability in Africa focused on the environment (Balehagn et al, 2021) and an article on enteric methane and the carbon footprint of beef and dairy cattle in the United States (Dillon et al., 2021). In addition, Johnson (2021) provides a table of 10 issues of Animal Frontiers that contain articles pertaining to sustainability in animal agriculture.

The lead article in this issue by Vonderohe et al. (2022) starts by asking the big question - how sustainable is sustainability? While there is a tendency to treat sustainability as a single entity that can heroically solve all of our problems going forward. The reality is there are many pieces to the puzzle of sustainability and sustainability and swine production is only one portion of the global topic of sustainability, consequently as much as we would like our superhero Sustainability Swine to solve all global sustainability issues, it simply isn’t possible.

The remaining articles in this issue of *Animal Frontiers* break down a number of the major issues of sustainability and swine production and begin to discuss the current research being implemented to solve these issues. The article by Zijlstra and Beltrena (2022) focuses on the feeding of coproducts as a means to reduce feed costs and ultimately to contribute to sustainable swine production. Shurson and Urriola (2022) argue that sustainable pork production requires moving from a linear supply chain to a circular supply chain that can optimize all aspects of swine production to include nutrition, health, societal and economic factors to name a few. Nitrogen and phosphorus are critical nutrients for swine but in excess are damaging to our environment. Latrou et al. (2022) consider optimizing phosphorus and nitrogen utilization in the pig which balances intake to the nutritional needs of individual animals. Another piece of the swine sustainability puzzle is the health of animals. He et al. (2022) explore the role of microbes on swine health and discuss nutritional and chemical means of impacting post-weaning diarrhea. The perspectives section of this issue begins with Farmer (2022) which suggests a novel method to employ to reduce piglet mortality. This proposal provides a mechanism to increase mammary development in sows that improves milk yield in sows and enhances piglet survival, reducing the number of pigs needed to meet global pork demand.

Even though, we have become increasingly efficient and hence more sustainable in terms of how we produce a pound of pork, the worldwide demand for pork has increased. Therefore, we finish out the issue with a discussion by Yu and Jensen (2022) focused on the need for a multidisciplinary approach to balance increased global demand for total amount of pork with sustainability.

*Conflict of interest statement.* None declared.

## About the Authors

**John “Scott” Radcliffe** is a Professor and Assistant Head in the Department of Animal Sciences at Purdue University. He received his B.S., M.S., and Ph.D. from Virginia Tech and was briefly on the faculty in the Department of Animal and Poultry Sciences at Virginia Tech prior to moving to Purdue University. Dr. Radcliffe has two fundamental research interests: 1) environmental nutrition—how do we feed pigs to ensure the highest level of environmental sustainability? and 2) gut health. Within those two areas, Dr. Radcliffe’s work can be partitioned into four categories: 1) reduction of the environmental impact of production animal agriculture (primarily swine) through increasing the efficiency of nutrient utilization and reducing nutrient excretion, 2) use of animal models to study nutrient absorption/utilization in human disease states, 3) gut health and function, and 4) the immune response in food allergy development and food-related digestive disorders.

**James Sartin** received a Ph.D. in physiology with an emphasis in metabolic endocrinology. He is an Emeritus Professor at Auburn University and serves as the Editor in Chief of *Animal Frontiers*. He is a Past President of the American Society of Animal Science and a Past President of the World Association of Animal Production. He is a founder and first Editor of Domestic Animal Endocrinology and a past Editor of the *Journal Animal Science*, *Animal Frontiers*, and *Translational Animal Science*.
